# Uptake of glucose-conjugated MGMT inhibitors in cancer cells: role of flippases and type IV P-type ATPases

**DOI:** 10.1038/s41598-017-14129-x

**Published:** 2017-10-24

**Authors:** Karl-Heinz Tomaszowski, Nadja Hellmann, Viviane Ponath, Hiroyuki Takatsu, Hye-Won Shin, Bernd Kaina

**Affiliations:** 1 Department of Toxicology, University Medical Center, Obere Zahlbacher Strasse 67, D-55131 Mainz, Germany; 2 Institute for Molecular Biophysics, Johannes Gutenberg-University, Jakob Welder Weg 26, D-55128 Mainz, Germany; 3 Graduate School of Pharmaceutical Sciences, and, Kyoto University, Sakyo-ku, Kyoto, 606-8501 Japan

## Abstract

The DNA repair protein *O* ^6^-methylguanine-DNA-methyltransferase (MGMT) is a key determinant of cancer resistance. The MGMT inhibitors *O* ^6^-benzylguanine (O^6^BG) and *O* ^6^-(4-bromothenyl)guanine (O^6^BTG) failed to enhance the therapeutic response due to toxic side effects when applied in combination with alkylating chemotherapeutics, indicating a need of inhibitor targeting. We assessed MGMT targeting that relies on conjugating the inhibitors O^6^BG and O^6^BTG to ß-D-glucose, resulting in O^6^BG-Glu and O^6^BTG-Glu, respectively. This targeting strategy was selected by taking advantage of high demand of glucose in cancers. Contrary to our expectation, the uptake of O^6^BG-Glu and O^6^BTG-Glu was not dependent on glucose transporters. Instead, it seems that after membrane binding the conjugates are taken up via flippases, which normally transport phospholipids. This membrane binding is the consequence of the amphiphilic character of the conjugates, which at higher concentrations lead to the formation of micelle-like particles in aqueous solution. The unusual uptake mechanism of the conjugates highlights the importance of proper linker selection for a successful ligand-based drug delivery strategy. We also demonstrate that proteins of the P4-Type ATPase family are involved in the transport of the glucose conjugates. The findings are not only important for MGMT inhibitor targeting, but also for other amphiphilic drugs.

## Introduction

In many countries, cancer is the second most common cause of death after cardiovascular diseases. Despite progress in understanding of the mechanism of its pathogenesis and continuous improvement in anticancer drug development, cancer in a metastatic case is often not curable. Several mechanisms prevent the development of effective drug-based cancer treatments, such as ineffective drug concentration reaching the tumor, severe side effects caused by unselective tissue distribution, and acquired resistance of tumors^1,2^. The DNA repair protein *O* ^6^-methylguanine-DNA methyltransferase (MGMT) repairs alkylation in the O^6^ position of guanine in DNA, thus protecting normal tissue against the mutagenic, clastogenic, and cytotoxic effects of endogenously and exogenously formed *O* ^6^-alkylguanine adducts^3–6^. In tumors, MGMT is the key factor determining resistance against alkylating anticancer drugs that induce toxicity mainly by the DNA adducts *O* ^6^-methylguanine and *O* ^6^-chloroethylguanine^7,8^. Therefore, pharmacological inhibition of MGMT renders cancer cells more susceptible to the treatment with *O* ^6^-alkylating agents, which are still used in the therapy of many cancer types^9^. Despite the development of efficient MGMT inhibitors such as *O* ^6^-benzylguanine (O^6^BG) and *O* ^6^-(4-bromothenyl)guanine (O^6^BTG, lomeguatrib), the therapeutic outcome in clinical trials conducted so far was not improved. This could be due to unselective reaction of MGMT inhibitors throughout the body enhancing systemic side effects, such as hematotoxicity, which in turn requires dose reduction of the alkylating drug. Therefore, strategies for selective inhibition of MGMT in tumors are expected to enhance the therapeutic index of alkylation-based chemotherapy^10^.

A hallmark of cancer is metabolic reprogramming^11^. In 1956, Otto Warburg reported for the first time that cancer cells exhibit an increase in glycolytic activity under aerobic conditions (Warburg effect)^12^. Later on it was shown that this is caused by overexpression of glucose transporters and glycolytic enzymes in cancer cells^13,14^. The increased dependence of cancer cells on glycolysis for energy production offers opportunities for diagnosis and treatment. A well-known application in this context is the position emission tomography (PET) using the glucose derivate 2-[fluorine-18] fluoro-2-deoxy-D-glucose (FDG) as radiotracer. This technique has been established as a useful tool in the surveillance of many solid cancers^15^. In cancer therapy, which exploits the enhanced glucose uptake in tumors, the pharmacological inhibition of glucose uptake or activity of certain glycolytic enzymes represents a potential target^16^. Another clinical application is the conjugation of anticancer drugs to glucose molecules, taking advantage of high glucose uptake as a drug delivery strategy for targeting cancer cells. For example, the chemotherapeutic drug streptozotocin, which is a conjugate of glucose and the powerful methylating agent N-methyl-N-nitrosourea, is used for treating island cell carcinomas of the pancreas due to preferential uptake through the glucose transporter protein GLUT2^17^.

The goal of drug targeting is to enable selective delivery of drugs to the target tissue, while minimizing the accumulation in non-target sites, thus reducing the adverse effects and improving the therapeutic index. Accumulation of drugs in the target tissue can be achieved by the utilization of specific characteristics of the tissue, such as the appearance of particular cell types and the overexpression of certain antigens on the cell surface^18^. Promising targeted drug delivery systems require stability and adequate pharmacokinetic properties of the drugs, high selectivity to the targeted site as well as release of therapeutic effective concentration in the target tissue^19^. There are various strategies of drug targeting, including the direct application of drugs into targeted tissues and by incorporation of drug molecules into a carrier as nanoparticles^20^ or conjugation of drugs with antibodies or ligands^2,21^. However, the linkers required for connecting the different molecules may influence several functions of the drug in question such as stability, folding, biological function, and pharmacokinetic properties. Therefore, the selection of a suitable linker is an important area in drug design. Factors in this context include linker composition and length as well as the molecule position of the attached linker sites to preserve biological function^22,23^.

The ATP-binding cassette (ABC) transporter family is well known because of its critical role in mediating tumor cell resistance to a variety of anticancer drugs, including hydrophobic, hydrophilic, and amphiphilic molecules^1^. Additionally, together with the P4 subfamily of P-type ATPases (P4-ATPases), several ABC transporters are involved in ATP-driven transfer of phospholipids between the two leaflets of the plasma membrane: transfer towards the cytoplasmatic side is established by flippases while the inverse process is performed by floppases. Proteins of the subfamily of P4-ATPases are only present in eukaryotic organisms and their biological functions have been implicated in generating lipid asymmetry, in scavenging exogenous lipids, and in inducing membrane curvature. Recent studies indicate that P4-ATPases differ in their substrate specificities and mediate unidirectional transport of a multitude of substrates, including lysophospholipids and the anticancer molecules alkylphospholipids^24–26^.

**Figure 1:**
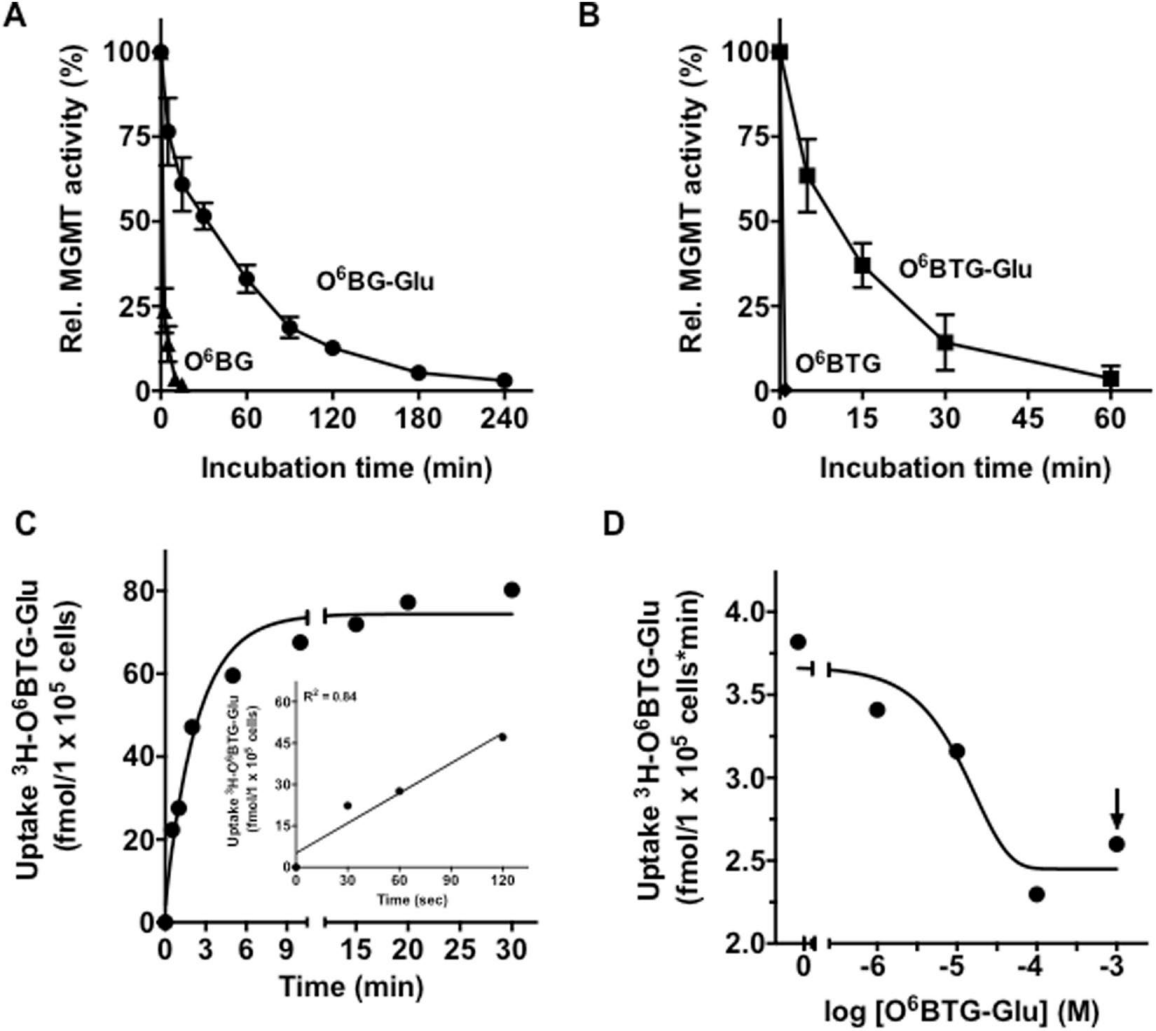
Kinetics of glucose-conjugated MGMT inhibitors. (**A**) T98G cells were treated with 10 μM O^6^BG (▲) and 25 μM O^6^BG-Glu (●) or (**B**) 5 μM O^6^BTG (◆) and 5 μM O^6^BTG-Glu. After indicated time points MGMT activity was determined. Data are from our previous work^28^ and shown here to demonstrate the efficiency of MGMT inhibition. (**C**) Time course of the uptake of ^3^H-O^6^BTG-Glu (47 nM) at 37 °C in Caco-2 cells. A monoexponential curve was fitted to the experimental data for graphic presentation. Insert: ^3^H-O^6^BTG-Glu uptake was linear for 120 s (r^2^ = 0.84). (**D**) Inhibition of ^3^H-O^6^BTG-Glu (5 nM) accumulation by increasing concentration of unlabeled O^6^BTG-Glu after 2 min incubation at 37 °C. The arrow indicates the concentration where a shift in turbidity of the aqueous solvent was observed. Each point represents the mean of three independent experiments.

Previously, we synthetized compounds, relying on conjugating of the MGMT inhibitors O^6^BG and O^6^BTG with a C8-linker to ß-D-glucose (the conjugates are O^6^BG-Glu and O^6^BTG-Glu respectively), in order to achieve an active targeting of cancer cells^27^. Although these glucose conjugates were efficient in inhibiting MGMT activity (Fig. 1A), their maximal sensitization of cells against alkylating agents was limited by cellular efflux mechanisms that reduce the actual intracellular concentration^28^. Here, we focused our studies on the mechanism of uptake of glucose-conjugated MGMT inhibitors. We show that the uptake of O^6^BG-Glu and O^6^BTG-Glu is  not dependent on glucose transporters, but relies on an unexpected mechanism of drug flipping. We further show that glucose conjugates are also taken up via P4-type ATPases and form larger particles in aqueous solution at higher concentrations. The data provide an example of how drug conjugates exert unexpected properties that determine their cellular uptake.

## Results

### Are glucose transporters involved in the uptake of glucose-conjugated MGMT inhibitors?

To demonstrate the ability of the conjugates to inhibit MGMT, T98G glioblastoma cells were treated with the MGMT inhibitors O^6^BG and O^6^BTG and with the glucose conjugates derived from them, O^6^BG-Glu and O^6^BTG-Glu, We observed a clear time-dependent inhibition of MGMT for the glucose-conjugated MGMT inhibitors. Complete inhibition of MGMT was achieved after 4 h for O^6^BG-Glu (Fig. 1A) and after 1 h for the more effective O^6^BTG-Glu (Fig. 1B). In contrast, the non-conjugated inhibitors caused MGMT inhibition already after 15 min for O^6^BG (Fig. 1A) and 1 min for O^6^BTG (Fig. 1B). The delayed inhibitory effect  of the glucose conjugates supports the hypothesis that uptake of the parental compound small-molecule inhibitors occurred via passive diffusion through the plasma membrane, while the glucose conjugates were taken up by active transport.

In the following set of experiments, we determined the rate of uptake of tritium labeled O^6^BTG-Glu (^3^H-O^6^BTG-Glu) using the cell line Caco-2. The human colon carcinoma cell line Caco-2 is a valuable *in vitro* system for glucose uptake studies because of the expression of various glucose transporters including facilitative glucose transporters (GLUTs) and sodium-dependent glucose transporters (SGLTs)^29–31^. Uptake of ^3^H-O^6^BTG-Glu was rapid and linear for at least 2 min (initial phase, 22 fmol/1 × 10^5^ cells/min), followed by a phase of slower uptake (Fig. 1C): between 15 and 30 min the rate is about 0.63 fmol/1 × 10^5^ cells/min. It is known that the glucose conjugates are substrates for ABC transporters, therefore the reduction of uptake rate with time could be caused by an active transport out of the cells, which has been shown previously^28^. In order to characterize further the initial uptake phase, the rate of uptake within the first 2 min was determined with ^3^H-O^6^BTG-Glu (5 nM) and increasing concentrations of the unlabeled conjugate (up to 1 mM). The rate decreased with increasing concentration of the unlabeled compound, indicating competition between labeled and unlabeled conjugate, which is typical for an active transport process with limiting number of interaction sites (Fig. 1D). Of note, further increasing the concentration of the conjugate (>1 mM O^6^BTG-Glu) caused a shift in turbidity of the aqueous solution, indicating particle formation (described below).

**Figure 2:**
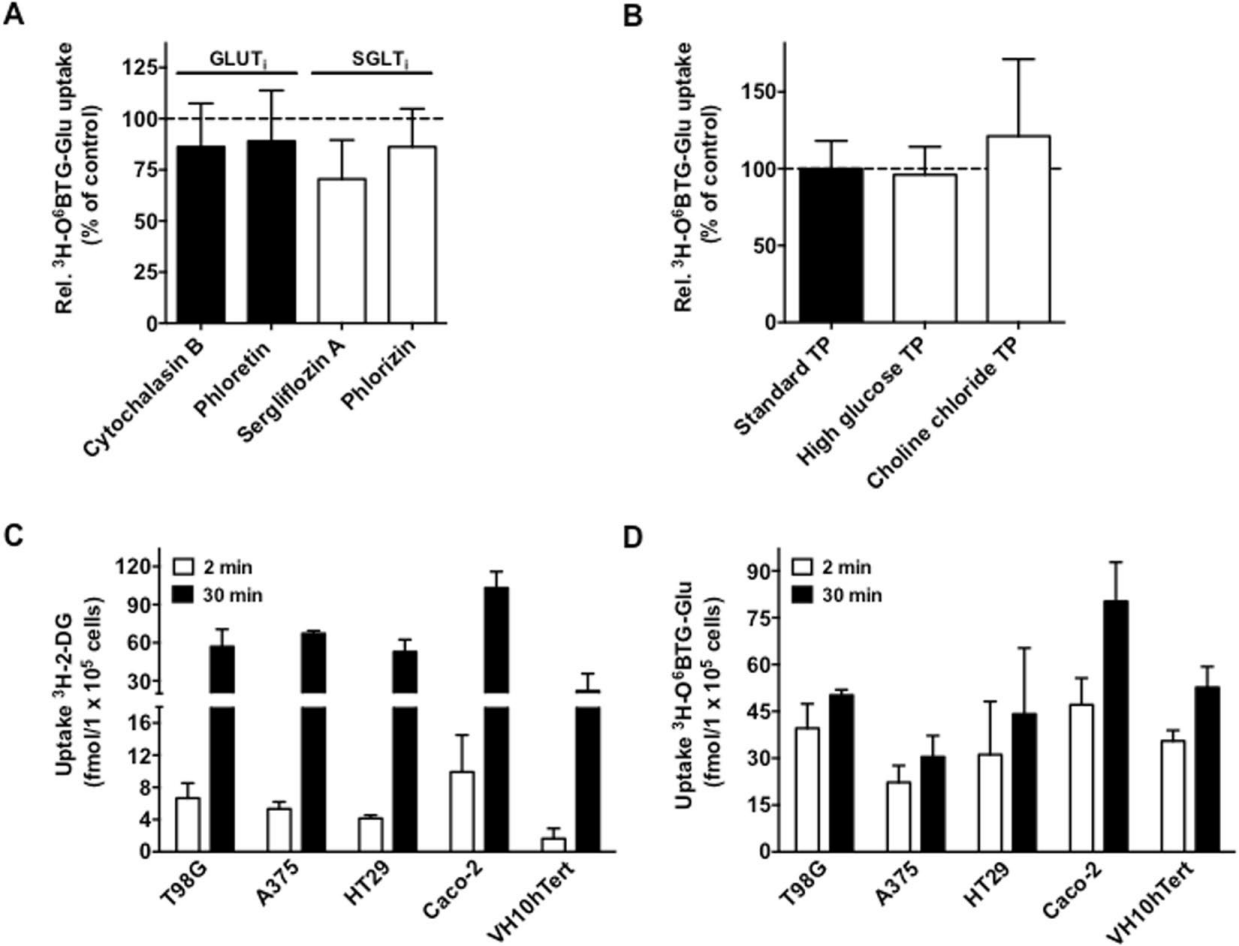
Effect of glucose transporters on ^3^H-O^6^BTG-Glu uptake. Uptake of ^3^H-O^6^BTG-Glu (47 nM) was determined in the presence of (**A**) various glucose transporter inhibitors or (**B**) different buffer conditions and expressed as percentage of control, defined as ^3^H-O^6^BTG-Glu uptake in standard transport buffer without inhibitors. Determination of (**C**) ^3^H-2-DG (3.75 nM) and (**D**) ^3^H-O^6^BTG-Glu (47 nM) uptake after 2 and 30 min in different types of cancer cell lines and a non-transformed fibroblast cell line. Details are described in text and material and methods section. All data are the mean of at least three independent experiments ± standard deviation (SD).

Next we set out to determine the uptake of ^3^H-O^6^BTG-Glu in the presence of known glucose transporter inhibitors and under different transport buffer conditions. As depicted in Fig. 2A, the GLUT inhibitors cytochalasin B and phloretin^32^ and the SGLT inhibitors phlorizin and sergliflozin A^33^ had only a slight if any effect on the uptake of ^3^H-O^6^BTG-Glu in Caco-2 cells. Furthermore, increasing the concentration of glucose, which would compete with ^3^H-O^6^BTG-Glu if GLUT or SGLT were involved, and the removal of sodium in the transport buffer had no effect on the conjugate uptake (Fig. 2B). We conclude that, contrary to our initial supposition, GLUT and SGLT transporters are not involved in the uptake of the glucose-conjugated MGMT inhibitors.

Measuring the effect of the repair inhibitor O^6^BG-Glu by means of the MGMT activity assay substantiated this conclusion. For the constructs the presence of glucose transporter inhibitors as well as the competition with glucose did not affect the inhibition of MGMT enzyme activity (Supplement Fig. S1). Additionally, we compared the accumulation of ^3^H-O^6^BTG-Glu and ^3^H-2-deoxy-D-Glucose (^3^H-2-DG) after short (2 min) and long (30 min) incubation periods in various human cancer cell lines to the one in the non-transformed human fibroblast cell line VH10hTert. As expected, cancer cells exhibited an enhanced uptake of tritium labeled glucose (^3^H-2-DG) compared to VH10hTert (about 5 to 9-fold higher after 2 min incubation compared to cancer cells) (Fig. 2C). In contrast, accumulation of ^3^H-O^6^BTG-Glu in VH10hTert cells was not generally lower than in cancer cells (Fig. 2D), suggesting a different uptake mechanism for glucose on one hand and glucose-conjugated MGMT inhibitors on the other. Long-time incubation (30 min) with ^3^H-O^6^BTG-Glu only slightly enhanced its uptake compared to 2 min incubation. As mentioned before this effect may be explained by our previous finding that glucose conjugates are a substrate for ABC transporters^28^. Collectively, these results show that glucose transporters are not involved in the uptake of the glucose-conjugated MGMT inhibitors O^6^BG-Glu and O^6^BTG-Glu.

### The amphipathic structure leads to particle formation of glucose conjugates

**Figure 3:**
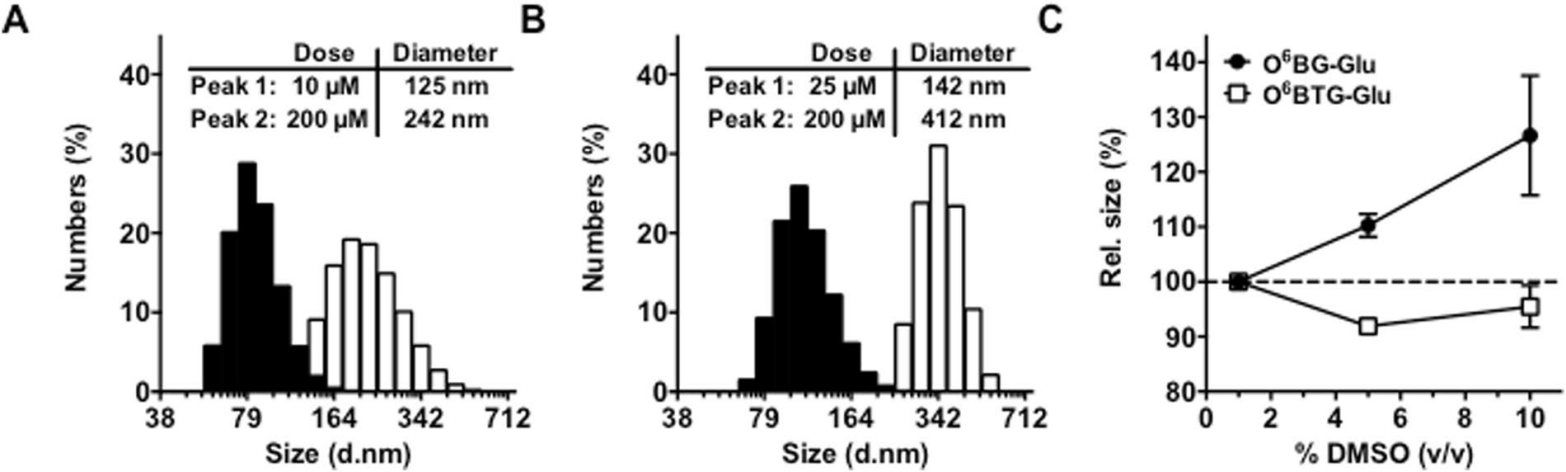
Glucose conjugates form particle in aqueous solution. Dynamic light scattering measurement was performed with different concentration (1–250 µM) of the glucose conjugates. Shown are two representative size distribution histograms (Peak 1: black bars, Peak 2: white bars) for O^6^BG-Glu (**A**) and O^6^BTG-Glu (**B**). Inserted tables present the mean diameter (n = 3) of the lowest concentration at which particles were detected (10 µM for O^6^BG-Glu and 25 µM for O^6^BTG-Glu) and at 200 µM for each glucose conjugate. (**C**) Relationship between DMSO concentration (v/v) and the particle size of 100 µM O^6^BG-Glu (●) and 100 µM O^6^BTG-Glu (□). All data are the average of three independent experiments ± SD.

Amphiphiles are chemical compounds possessing covalently bound hydrophilic and hydrophobic parts, e.g. detergents, surfactants, cholesterol, and lipids. Due to the hydrophobic effect this compounds form a variety of structures in aqueous solution^34^. The glucose-conjugated MGMT inhibitors used in this study also consist of a large hydrophobic part (the modified guanine base with the C8-linker) and a hydrophilic part (the glucose), suggesting that the conjugates might have the ability of self assemblance. The first indication that glucose conjugates form larger particles in aqueous solution came from a shift in turbidity at high concentration (1 mM), which resulted from precipitates in the solution. To determine more precisely whether the glucose conjugates form particle-like structures at a lower concentration than 1 mM, we performed dynamic light scattering measurements over a concentration range of 1–250 µM. Both O^6^BG-Glu (Fig. 3A) and O^6^BTG-Glu (Fig. 3B) form particles with narrow size distribution (polydispersity index < 0.3) and an average diameter size of about 140 to 400 nm in solution, depending on the concentration and the glucose conjugate. Interestingly, although the glucose conjugates are very similar in chemical structure, differing only in the benzyl- and 4-bromothenyl group at the O^6^-position of guanine, they posses a clear difference in their ability to form particles. The concentration for obtaining measurable particles is lower in case of O^6^BG-Glu (~10 µM) compared to O^6^BTG-Glu (~25 µM), indicating differences in the critical micelle concentration (CMC) for the glucose conjugates. Of note, using different DMSO concentrations in the solvent, we observed an increase in particle size for O^6^BG-Glu whereas the particles formed by O^6^BTG-Glu remained unaffected (Fig. 3C). The results support the notion that the amphiphilic properties of the glucose conjugates lead to the formation of micelle-like particles in aqueous solution. This presumably reduces the number of active monomers of O^6^BG-Glu and O^6^BTG-Glu taken up by the cell.

### Is there an active uptake mechanism for the amphiphilic conjugates and do they interact with the plasma membrane?

**Figure 4:**
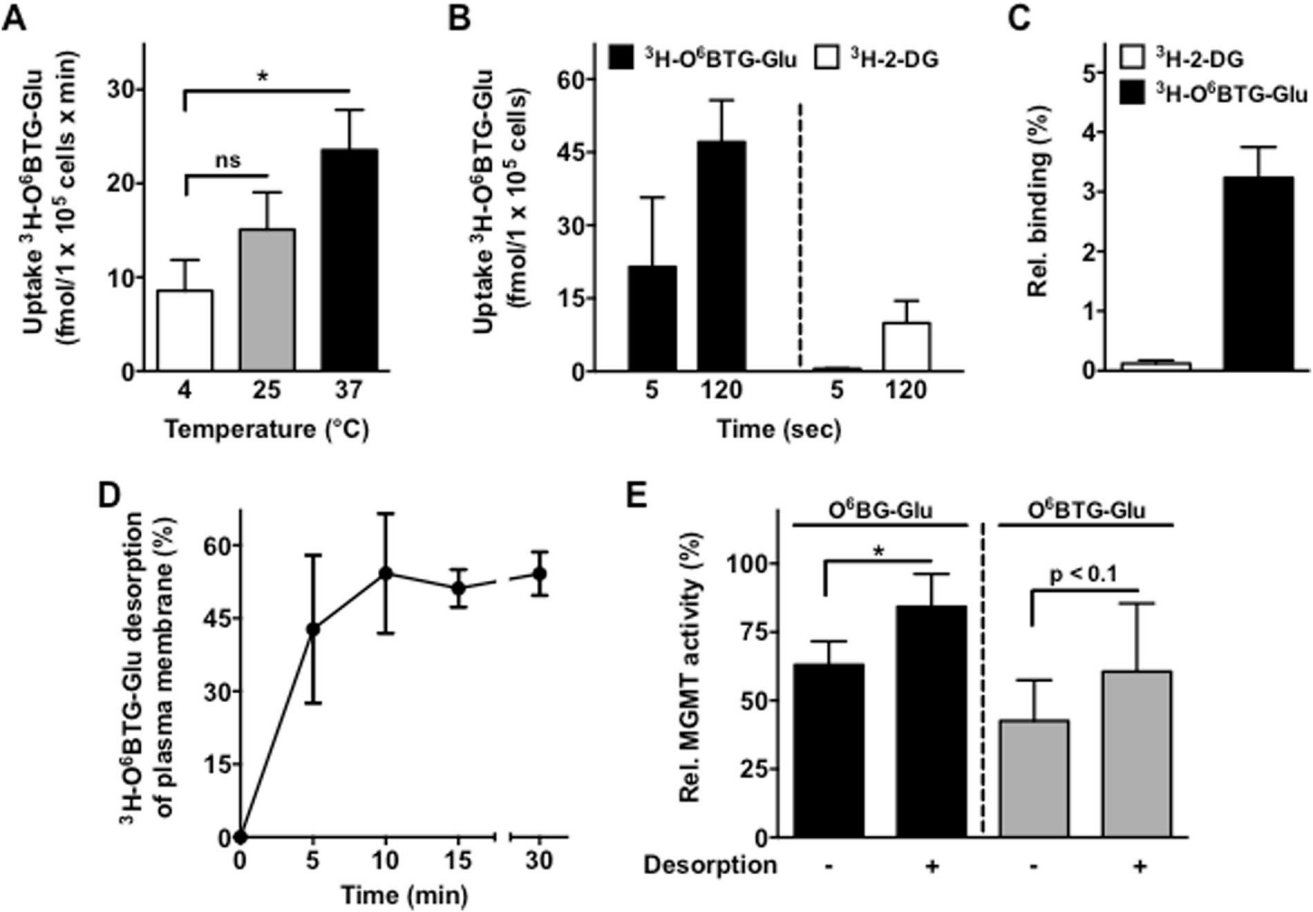
Direct adsorption of glucose conjugates through the plasma membrane. (**A**) Uptake of ^3^H-O^6^BTG-Glu (47 nM) at different incubation temperatures in Caco-2 cells. (**B**) Accumulation of ^3^H-O^6^BTG-Glu (47 nM) and ^3^H-2-DG (3.75 nM) after 5 and 120 seconds (sec) at 37 °C in Caco-2 cells. (**C**) Membrane binding of ^3^H-O^6^BTG-Glu (5 nM) and ^3^H-2-DG (4 nM) to multilamellar vesicles (MLV) after 5 sec incubation. (**D**) To determine the amount of adsorbed ^3^H-O^6^BTG-Glu in the plasma membrane Caco-2 cells were incubated with ^3^H-O^6^BTG-Glu (47 nM) for 30 min at 4 °C. Afterwards, 10 mg/ml BSA was added and cells were incubated for indicated time intervalls at 4 °C to allow desorption and BSA-binding of externally bound conjugate. The desorption buffer also incorporated ABC transporter inhibitors (10 µM tariquidar, 10 µM MK571, 5 µM Ko143) to block the active efflux by various multidrug transporters. (**E**) T98G cells were incubated with 25 µM O^6^BG-Glu or 5 µM O^6^BTG-Glu for 15 min at 37 °C. Afterwards, cells were subjected to desorption phase for 10 min at 4 °C as described in (**D**). Details are described in text and material and methods section. All data represents the mean +/− SD. *p < 0.05. ns: not significant.

At concentrations below 10 µM, the glucose conjugates are soluble and do not form measurable amounts of particles. It is therefore reasonable to conclude that in this low concentration range, in which MGMT can significantly be inhibited^27^, transporter mediated influx is a major mechanism of uptake. The human genome expresses more than 400 transporter proteins, whose substrate specificities are not known in detail^35^. To clarify whether transporter proteins are involved in the uptake of our glucose conjugates, we determined the transport rate of ^3^H-O^6^BTG-Glu at different temperatures, keeping in mind that passive diffusion occurs at low temperatures, whereas active uptake usually does not. In Fig. 4A, we demonstrate that the uptake of ^3^H-O^6^BTG-Glu was clearly temperature-dependent with the highest uptake rate at 37 °C. However, at 4 °C a high amount of radioactivity was still detected in association with the cells (about 40% of the radioactivity measured at 37 °C). A reasonable explanation for the high amount of radioactivity at 4 °C could be a direct binding of the conjugate on the plasma membrane. Indeed, compared to ^3^H-2-DG, the conjugate ^3^H-O^6^BTG-Glu showed a high amount of radioactivity even after 5 seconds of incubation with Caco-2 cells (Fig. 4B) as well as following incubation with artificial membrane vesicles (Fig. 4C). We conclude that the glucose conjugates have the property to adsorb to the plasma membrane.

Further, we analyzed whether the observed adsorption influenced the uptake into the cells. To this end, we performed desorption experiments. The desorption buffer contained ABC transporter inhibitors for blocking the active efflux by multidrug pumps. It also contained BSA that facilitates the removal of loosely bound conjugates from the membrane. After incubation of membrane bound ^3^H-O^6^BTG-Glu in the desorption buffer at 4 °C, nearly 55% of the measured radioactivity was lost after 10 min and did not further increase at longer desorption times (Fig. 4D). Also the MGMT inhibition by both O^6^BG-Glu and O^6^BTG-Glu was reduced if incubation for 15 min was followed by a desorption phase (Fig. 4E). Collectively, this data supports the notion that both glucose conjugates possess binding capacity to the plasma membrane, probably due to their amphiphilic properties.

### Is uptake mediated by a flip-flop mechanism or by P4-Type ATPases?

**Figure 5:**
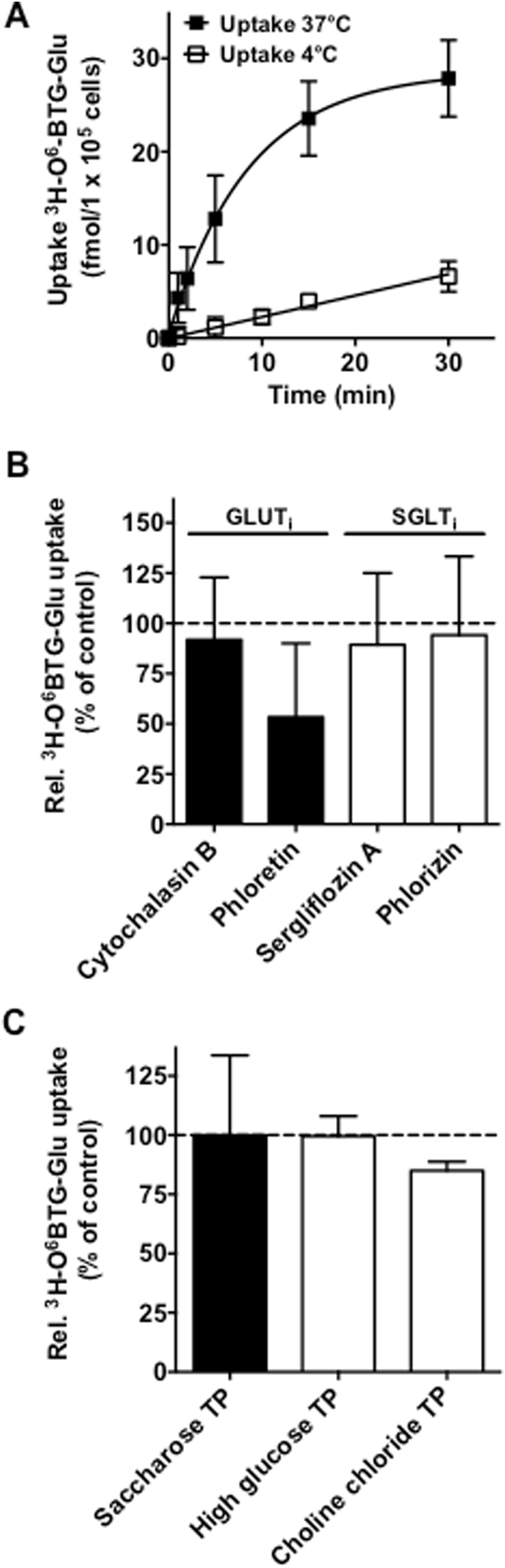
Residual accumulation of ^3^H-O^6^BTG-Glu in Caco-2 cells after removal of plasma membrane bound ^3^H-O^6^BTG-Glu and the effect of glucose transporter inhibitors. (**A**) Time course of the uptake of ^3^H-O^6^BTG-Glu (24 nM) at 4 °C and 37 °C. After incubation for indicated time intervals,  a desorption phase (see legend Fig. 4) was incorporated in the protocol in order to remove plasma membrane bound ^3^H-O^6^BTG-Glu. A monoexponential uptake curve was fitted to the experimental data. Subsequently, uptake of ^3^H-O^6^BTG-Glu (24 nM) for 5 min at 37 °C was determined in the presence of (**B**) various glucose transporter inhibitors or (**C**) different buffer conditions and expressed as percentage of control, defined as ^3^H-O^6^BTG-Glu uptake in standard transport buffer without inhibitors. Each point represents the mean of three independent experiments +/− SD.

Taking into account that the glucose conjugates show considerable affinity to the plasma membrane, we examined the involvement of flippases. First, we compared the kinetics of ^3^H-O^6^BTG-Glu uptake after removal of unspecific binding to the plasma membrane. As shown in Fig. 5A, the transport rate of ^3^H-O^6^BTG-Glu was time-dependent and the accumulation of the conjugate was clearly higher at 37 °C than at 4 °C. Within 30 min a level of 28.6 +/− 2.8 fmol/10^5^ cells was reached. However, also at 4 °C the glucose conjugate showed an increase in accumulated substance with a constant rate (r^2^ = 0.89). It is unclear whether the continuous accumulation in this case is caused by uptake by passive diffusion into the cell or reflects merely binding to the plasma membrane. Nevertheless, the difference between the low-temperature uptake (4 °C) and the high-temperature uptake (37 °C) can be attributed to an active transport mechanism. Subsequently, experiments were performed (5 min incubation at 37 °C) in the presence or absence of glucose transport inhibitors including a desorption phase to remove unspecifically bound conjugates from the plasma membrane. As shown in Fig. 5B, pretreatment with phloretin reduced the ^3^H-O^6^BTG-Glu accumulation, whereas sergliflozin A and phlorizin had no effect. The same was found upon addition of high glucose concentrations and upon replacing sodium with choline in the incubation buffer (Fig. 5C). Since all of these treatments have no effect on the uptake of ^3^H-O^6^BTG-Glu, the data strongly confirm what has been concluded earlier on the basis of MGMT inhibition and support the notion that glucose transporters, including GLUT and SGLT, are not involved in the uptake of the glucose conjugates.

**Figure 6:**
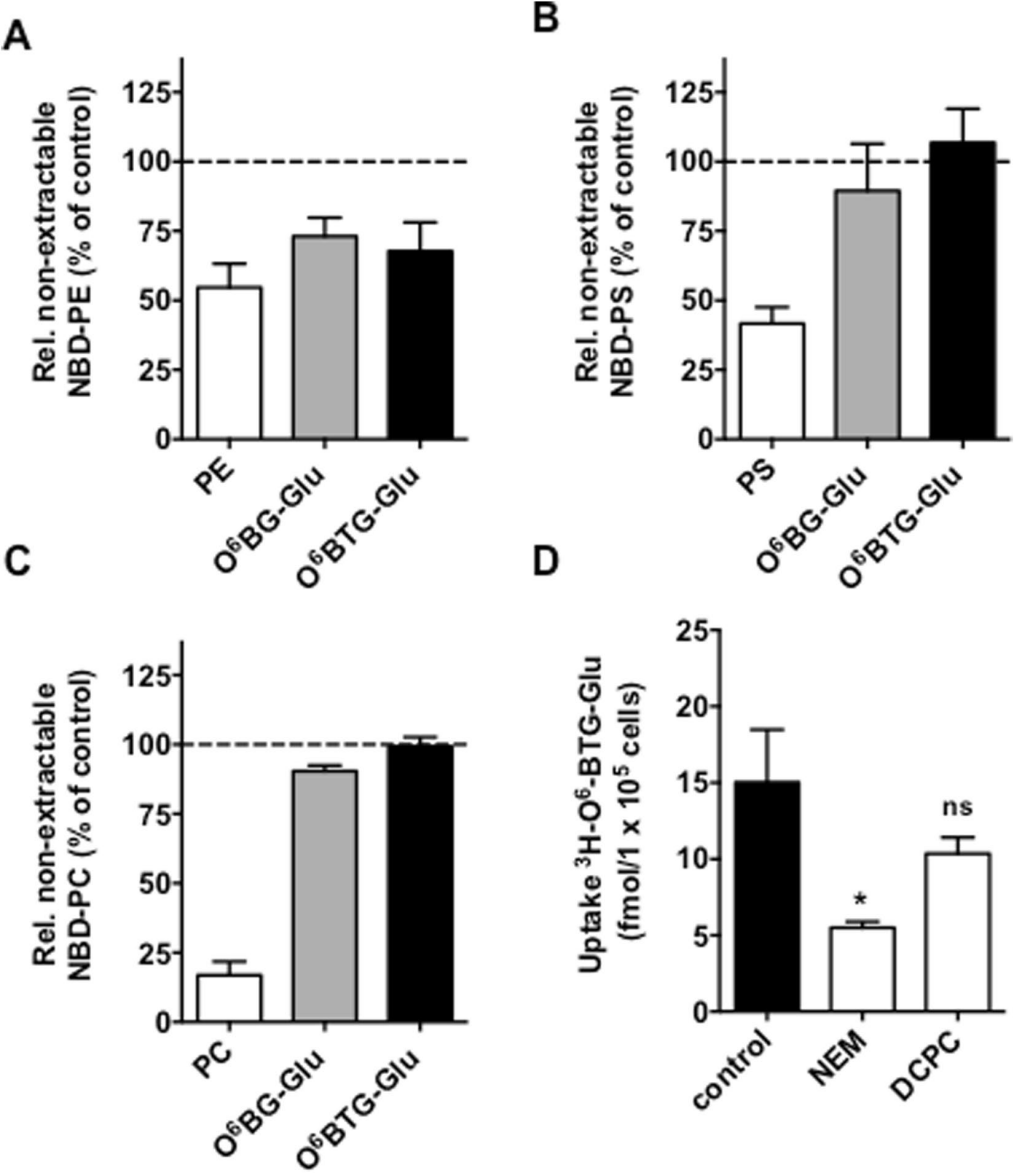
Influence of flippases (P4-Type ATPases) on the intracellular transport of the glucose conjugates. T98G cells were incubated with (**A**) 0.2 µM NBD-PE, (**B**) 0.5 µM NBD-PS or (**C**) 0.2 µM NBD-PC for 15 min at 15 °C in presence of 10 µM O^6^BG-Glu, O^6^BTG-Glu or the respective non-fluorescent phospholipid. Afterwards, cells were subjected to desorption phase in order to extract NBD-lipids adsorbed on the exoplasmatic leaflet of the plasma membrane. Translocated NBD-lipids into the cytoplasmic leaflet of the plasma membrane was determined by flow cytometry. (**D**) Uptake of ^3^H-O^6^BTG-Glu (24 nM) after 5 min at 37 °C following preincubation with 5 mM Diethylpyrocarbonate (DCPC) or N-ethylmaleimide (NEM). Significant difference to untreated control were calculated by one-way ANOVA test. *p < 0.05. ns: not significant. All data are the average of at least three independent experiments +/− SD.

Because of the amphiphilic properties of the glucose conjugates, we pondered the possibility that flippases (P4-Type ATPases) might be involved in the intracellular transport of the glucose conjugates. To check  this hypothesis, we analyzed the accumulation of externally added fluorescent phospholipid analogues NBD-PC, NBD-PE and NBD-PS at 15 °C (at this temperature endocytosis is minimal while active uptake is still working) in the glioma cell line T98G. Lipids were added in the absence and presence of the glucose conjugates, and fluorescent phospholipids that were not incorporated, but still present in the outer plasma membrane, were removed employing fatty-acid-free BSA. Non-fluorescent phospholipids instead of the conjugates were used as a positive control for demonstrating the competition effect. As shown in Fig. 6A, both O^6^BG-Glu and O^6^BTG-Glu reduced the internalization of NBD-PE to a similar extent as the non-fluorescent substrate PE. In contrast, the glucose conjugates showed no competition effect on either NBD-PS or NBD-PC (Fig. 6B and C), suggesting that reduced uptake due to the presence of the conjugates is specific for PE, resulting from competition for the same uptake mechanism. The transport by flippases of phospholipids is vulnerable to cysteine and histidine modification reagents such as N-ethylmaleimide (NEM) and diethylpyrocarbonate (DEPC), respectively^36^. As shown in Fig. 6D, pretreatment with NEM resulted in nearly 60% reduction, and pretreatment with DEPC in 25% reduction of ^3^H-O^6^BTG-Glu intracellular accumulation. Collectively, the data strongly suggests that the intracellular uptake of O^6^BTG-Glu is mediated by flippases (P4-type ATPases).

### Which P4-Type ATPase is responsible for uptake?

**Figure 7:**
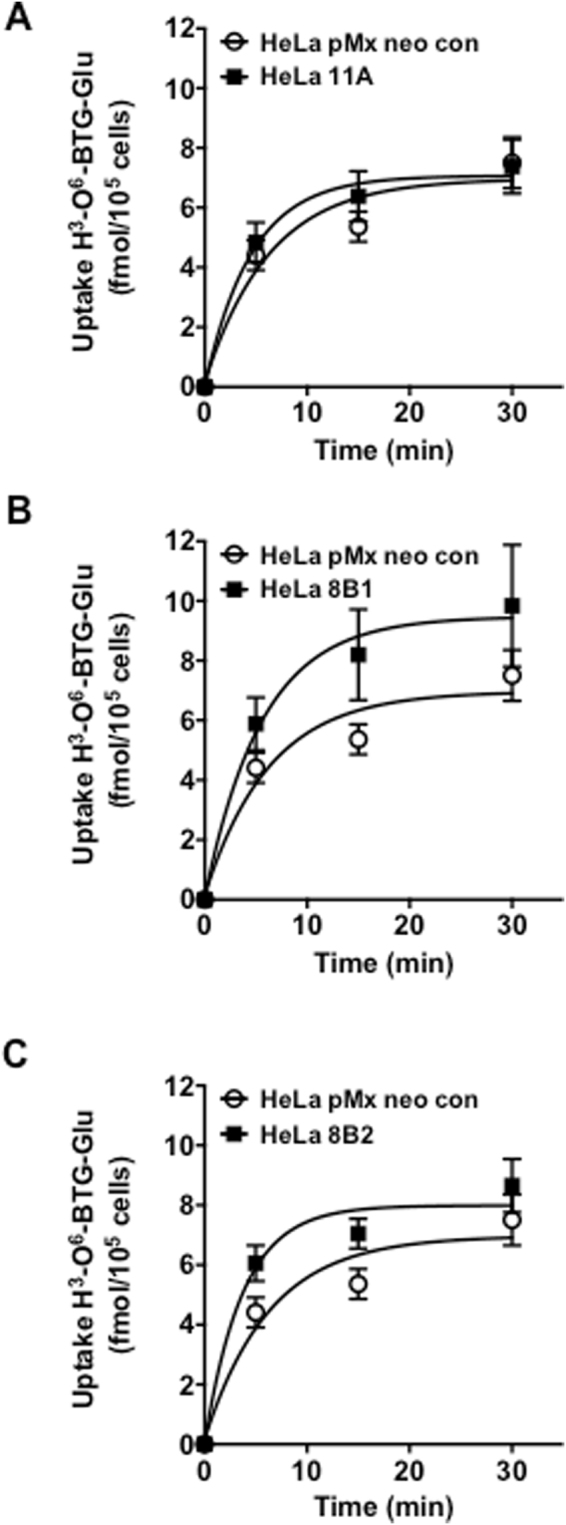
Transporter-mediated uptake of ^3^H-O^6^BTG-Glu by P4-type ATPases. Time course of the uptake of ^3^H-O^6^BTG-Glu (24 nM) at 37 °C in HeLa cells stably overexpressing human P4-type ATPases (**A**) ATP11A (HeLa11A), (**B**) ATP8B1 (HeLa 8B1) and (**C**) ATP8B2 (HeLa 8B2) and their parental control cell line (designated as HeLa pMx neo con). The  incubation for indicated time intervals was followed by a desorption phase in order to remove plasma membrane bound ^3^H-O^6^BTG-Glu. Data show the mean +/− SEM. Lines are included to guide the eye.

Members of the P4-type ATPase are expressed in human cells, mediating the transport of phospholipids from the exoplasmic to the cytoplasmic leaflets of cellular membranes^25^. In order to evaluate the substrate specificity of human P4-type ATPases for O^6^BTG-Glu, we used HeLa cells stably overexpressing either ATP11A, ATP8B1, or ATP8B2. As shown in Fig. 7A, in parental HeLa cells (designated as HeLa pMx neo con) ^3^H-O^6^BTG-Glu accumulation increased in a time-dependent manner with a level of 7.0 +/− 0.7 fmol/10^5^ cells at 30 min, potentially mediated by endogenous flippases. HeLa cells overexpressing ATP11A were able to take up the same amount as the isogenic parental cells. In contrast, the level of ^3^H-O^6^BTG-Glu incorporation was consistently higher in cells stably expressing ATP8B1 compared to the parental HeLa cell line with a level of 9.5 +/− 1.2 fmol/10^5^ cells at 30 min (Fig. 7B). The same was true for ATP8B2 transfected cells, with a level of 8.0 +/− 0.5 fmol/10^5^ cells at 30 min (Fig. 7C). The data support the notion that ATP8B1 and ATP8B2 are involved in uptake of the MGMTinhibitor conjugates. To further substantiate the data, we downregulated ATP8B1 in HeLa cells by siRNA. Treatment with O^6^BG-Glu resulted in inhibition of MGMT, which was reproducibly lower in ATP8B1 knockdown than in cells treated with control siRNA (the difference was at the border of significance, which is likely due to incomplete downregulation by the siRNA approach; Supplement Fig. S2). Taken together, the available data supports the notion that the uptake of glucose-conjugated MGMT inhibitors is mediated by P4-type ATPases without the involvement of the classical glucose transporters GLUT and SGLT.

## Discussion

Drug targeting is a promising strategy in cancer therapy. Targeting of drugs enhances the therapeutic outcome by improving the specificity of delivery while reducing side effects due to damage of healthy tissues. Under the various possibilities to achieve selective accumulation in targeted tissue, ligand-based drug delivery is a reasonable strategy. In this case, the drug of interest requires coupling to a targeting moiety, either directly or through a carrier that displays high affinity to specific components in the target tissue. Numerous molecules have been used for this purpose such as antibodies, ligands for receptors, peptides or small molecules including vitamins and sugars^2^. Despite continuous progress in this field over the last several decades, only a few drug delivery systems have been successful in clinical studies. There are several reasons that complicate the clinical translation of drug targeting systems including, but not limited to, the lack of homogeneity of the targeted tissue, complexity of the biological environment, and the high number of variables influencing the design and fate of drug delivery systems such as selection of targeting moieties and coupling strategies.

In this study, we have investigated glucose-conjugated MGMT inhibitors as a targeting strategy for MGMT positive cancers, which are inevitably resistant to alkylating drug-based therapies^4^. It is known that tumor cells possess higher glucose consumption than normal cells and overexpress certain glucose transporters, such as GLUT1. Therefore, to achieve selective targeting of cancer cells, we conjugated the potent MGMT inhibitors O^6^BG and O^6^BTG to ß-D-glucose with a hydrophobic C8-spacer^37^. The C8-linker between the glucose and MGMT inhibitor was required to preserve the MGMT inhibitor activity of the glucose conjugates *in vitro* and in intact cells and to enhance the toxic effects of O^6^-alkylating anticancer drugs^27^. Previously, we have also shown that the glucose conjugates are transported out of the cell by multidrug efflux transporters^28^. However, the mechanism of uptake was not yet elucidated.

Here we show that, contrary to our expectation, the glucose conjugates are not subject to transport by the glucose transporters GLUT and SGLT. Neither addition of high glucose concentrations nor the presence of glucose transporter inhibitors considerably affected the uptake of tritium labelled O^6^BTG-Glu. It also did not impact the MGMT inhibition in cells exposed to O^6^BG-Glu. Several strategies were reported in which glucose was linked to anticancer drugs such as alkylating agents and taxanes^38^, but convincing evidence for selective uptake was not presented. The slight effect of phloretin observed upon co-treatment with glucose-conjugated MGMT inhibitors should be interpreted with caution: phloretin was reported to have side effects on cells, e.g. modification of the property of lipid bilayers and reducing the cellular ATP level^39,40^. Therefore, phloretin may affect various uptake mechanisms that are dependent on ATP and the integrity of the plasma membrane.

Conjugation to glucose is expected to improve the water solubility of hydrophobic drugs. However, the need to introduce a spacer between glucose and drug can lead to unintended properties. The use of a hydrophobic C8-linker leads to amphiphilic molecules with new properties such as direct binding in/on the plasma membrane and self-assembly in aqueous solution. The molecular structure of O^6^BG-Glu and O^6^BTG-Glu is not in accordance with classical micelle- and bilayer forming, amphiphilic molecules (hydrophilic head group and hydrophobic tail), but rather is barbell-like with one hydrophilic and one hydrophobic bulky head group connected by a hydrophobic linker. For this reason, it is unknown which shape of particles are produced by the glucose conjugates. Based on our preliminary data, it seems that the glucose conjugates O^6^BG-Glu and O^6^BTG-Glu have different physico-chemical properties with respect to particle formation such as CMC, particle size, and possibly shape. It is conceivable that particle formation of the glucose conjugates has an influence on its uptake. While the particles probably can not be transported by selective transporters, it is known that particles can be taken up  into cells by endocytosis. Thus, depending on the relative amount of monomers and particles, which in turn depends on the conjugate concentration, the contribution of selective and passive transport such as endocytosis may change. In addition to particle formation, the hydrophobic linker for coupling O^6^BG and O^6^BTG to glucose itself may play a major role in the uptake of the glucose conjugates. The hydrophobic C8-linker seems to cause some structural similarity between the glucose conjugates and membrane phospholipids despite the bulky hydrophobic headgroup. This is support by their capacity to block the transport of the fluorescent phospholipids NBD-PE in a competition assay. Furthermore, we showed that HeLa cells stably overexpressing the flippases ATP8B1 and ATP8B2 accumulate higher amounts of ^3^H-O^6^BTG-Glu than their parental cell line. These transport proteins are known to be involved in inward phospholipid transport of phosphatidylcholine^41^, phosphatidylserine and phosphatidylethanolamine^42,43^ from the exoplasmatic leaflet to cytoplasmatic leaflet of biological membranes, suggesting that our glucose conjugates, O^6^BG-Glu and O^6^BTG-Glu, compete with natural phospholipids to enter the cell by the same transport proteins. Knowledge concerning the mechanism of substrate translocation by P4-Type ATPases across the cellular membrane is still limited, however, it has been reported that the relative size of substrates transported is an important factor. Size and location of hydrophilic and hydrophobic moieties are almost identical for the glucose conjugates O^6^BG-Glu and O^6^BTG-Glu and indeed their capacity to reduce NBD-PE uptake is similar., It should be noted that we cannot exclude the possibility that O^6^BG-Glu and O^6^BTG-Glu are additionally transported by other uptake mechanisms for amphiphilic molecules such as protein-independent transbilayer transport, endocytosis, or transporter-mediated uptake by the organic anion-transporting polypeptide (OATP) familiy^35,44,45^., which indicates a complex scenario that has to be taken into account in drug targeting. 

## Methods

### Cell lines and chemicals

The melanoma cell line A375, the cervix adenocarcinoma cell line HeLa S3 and the glioma cell line T98G were purchased from American Type Culture Collection (ATCC), the colon carcinoma cells Caco-2 and HT29 from Cell Lines Services (Heidelberg, Germany). The SV40T-immortalized human foreskin fibroblast cell line VH10hTert were a kind gift of Prof. Mullenders (Institute of Toxicogenetics, Leiden, Netherlands). HeLa cells stably overexpressing human P4-type ATPases (ATP11A, ATP8B1 and ATP8B2) and their parental control cell line were provided by Dr. Hye-Won Shin. For selection, these cell lines were routinely cultured in media containing 1.0 mg/ml G418, but the selective agents were omitted during the experiments. All cell lines were maintained in appropriate media supplemented with 10% fetal calf serum (FCS), 10 U/ml penicillin, and 10 mg/ml streptomycin. The synthesis of the glucose-conjugated MGMT inhibitors has been described previously^37^.

The glucose-conjugate 2-amino-6-(benzyloxy)-9-(octyl-ß-D-glycosyl)-purine (O^6^BG-Glu) was kindly provided by Prof. Schirrmacher (Montreal Neurological Hospital and Institute, Montreal, Canada). The second glucose-conjugated MGMT inhibitor 2-amino-6-(4-bromothiophen-2-yl-methoxy)-9-(octyl-ß-D-glycosyl)-purine (O^6^BTG-Glu) was purchased from Haoyuan Chemexpress Co., Limited (Shanghai, China). Preparation of tritium labeled O^6^BTG-Glu (^3^H-O^6^BTG-Glu, with specific activity of 8 Ci/mmol) was performed by Biotrend (Cologne, Germany) and the purity was verified by HPLC. Tritium labeled 2-deoxy-D-Glucose (^3^H-2-DG) was purchased from PerkinElmer (Rodgau, Germany). Sergliflozin A was purchased from Kissei Pharmaceutical (Japan), Elacridar and Tariquidar from MedKoo Biosciences (North Carolina, USA). The MGMT inhibitor *O* ^6^-benzylguanine (O^6^BG) was purchased from Sigma Aldrich (Munich, Germany) and *O* ^6^-(4-bromothenyl)guanine (O^6^BTG) was kindly provided from Dr. Geoff Margison (Paterson Institute for Cancer Research, Manchester, U.K.). The NDB labeled phospholipids were purchased from Avanti Polar Lipids (Alabaster, USA). The following lipids were used: 1-oleoyl-2-[6-[(7-nitro-2–1,3-benzoxadiazol-4-yl)amino]hexanoyl]-sn-glycero-3-phosphoserine (NBD-PS), 1-oleoyl-2-[6-[(7-nitro-2-1,3-benzoxadiazol-4-yl)amino]hexanoyl]-sn-glycero-3-phosphoethanolamine (NBD-PE), 1-oleoyl-2-[6-[(7-nitro-2-1,3-benzoxadiazol-4-yl)amino]hexanoyl]-sn-glycero-3-phosphocholine (NBD-PC). All other chemicals were obtained from Sigma-Aldrich (Munich, Germany).

### Uptake experiments

For all experiments, cells were seeded in 6-well plates two days prior to transport experiments. On the day of the experiment, the cell monolayers were washed and preincubated at 37 °C for at least 15 min using Modified Hank’s balanced salt solution (HBSS): 0.952 mM CaCl_2_, 5.36 mM KCl, 0.441 mM KH_2_PO_4_, 0.812 mM MgSO_4_, 136.7 mM NaCl, 0.385 mM Na_2_HPO_4_, 1 mM glucose, 10 mM HEPES, pH = 7.4 (referred to as standard transport buffer (TP) in the following). A few uptake experiments were performed using other transport buffers, which include the same composition with the following modification: High glucose TP: increase of glucose concentration to 25 mM; choline chloride TP: 136.7 mM choline chloride instead of NaCl; saccharose TP: 10 mM saccharose and 0.1 mM glucose instead of 1 mM glucose. If not stated otherwise, transport inhibitors were also present during both preincubation and incubation period: 10 µM cytochalasin B, 500 µM phloretin, 100 µM sergliflozin A, 100 µM phlorizin. To determine the transport rate, cells were incubated with respective transport buffer containing ^3^H-O^6^BTG-Glu (333 µCi/ml, 47 nM) and 1 µM unlabeled O^6^BTG-Glu (or 67 µCi/ml ^3^H-2-DG, 4 nM) at 37 °C for indicated time points. Uptake was stopped by rinsing cells three times with ice-cold glucose transport buffer. Afterwards cells were solubilized in 1 M NaOH and the cell lysates were neutralized using 1 M HCl. The radioactivity was measured by liquid scintillation counting (Canberra Packard, Dreieich, Germany). Competition by the unlabeled glucose-conjugate O^6^BTG-Glu was determined using Caco-2 cells grown in 6-well plates. Cells were incubated with a solution containing ^3^H-O^6^BTG-Glu (33 µCi/ml, 5 nM) and 0–1000 µM of unlabeled O^6^BTG-Glu (before added to transport buffer, the tritium labeled and unlabeled conjugate were mixed in a hydrophobic solution) at 37 °C for 2 min. The uptake of ^3^H-O^6^BTG-Glu was analyzed using scintillation counter as described above. In order to examine the influence of direct adsorption onto the plasma membrane on ^3^H-O^6^BTG-Glu uptake, an additional desorption phase was incorporated. The cells were incubated with transport buffer containing ^3^H-O^6^BTG-Glu (167 µCi/ml, 24 nM) and 1 µM unlabeled O^6^BTG-Glu at 37 °C for indicated time intervals. After the washing steps, the cells were subjected to desorption phase using 10 mg/ml bovine serum albumin (BSA) in ice-cold glucose transport buffer for 10 min at 4 °C, in order to remove the excess of ^3^H-O^6^BTG-Glu adsorbed on the outer plasma membrane. The desorption buffer also incorporated ABC transporter inhibitors (10 µM tariquidar, 10 µM MK571, 5 µM Ko143) to block the active efflux by various multidrug transporters. The uptake of ^3^H-O^6^BTG-Glu was analyzed using scintillation counter as described above. Diethylpyrocarbonate (DCPC) and N-ethylmaleimide (NEM), a specific modifier of histidine and cysteine residues, respectively, was used to examine the contribution of flippases to ^3^H-O^6^BTG-Glu uptake. The cells were preincubated with transport buffer containing 5 mM DCPC and/or 5 mM NEM for 15 min at 37 °C. After removing of the transport buffer, cells were washed and treated as described above.

### Binding assay on liposomes

Preparation of multilamellar vesicles (MLV), consisting of phosphatidylcholine and cholesterol, were performed based on the method of Budai *et al*.^46^. For binding experiments 2.5 mg MLVs were mixed with ^3^H-O^6^BTG-Glu (33 µCi/ml, 5 nM) or ^3^H-2-DG (67 µCi/ml, 4 nM) in appropriate transport buffer (500 µl) for 5 sec. Afterwards, MLVs were centrifuged (14.000 rpm, 10 min, 4 °C) and washed three times with ice-cold glucose transport buffer. Radioactivity of samples was determined by liquid scintillation counting and the “bound fraction” was calculated as difference between “total inserted” and “bound MLVs” radioactivity.

### MGMT activity assay

Determination of MGMT activity was performed as recently published^28^. After treatment, cell extracts were prepared and the specified amount of protein extract was incubated with tritium labeled thymus DNA. After precipitation of proteins, the radioactivity was determined. MGMT activity was expressed as fmol of [^3^H]-methyl transferred from radioactively labeled DNA to MGMT protein per mg of total protein extract.

### Flippase assay

Incorporation of NBD-phospholipids was analyzed by flow cytometry as described^41^. In brief, T98G cells were detached from dishes, collected in phosphate buffered saline (PBS) containing 5 mM ethylenediaminetetraacetic acid (EDTA), and then centrifuged (4 min, 4 °C, 4.000 rpm). Cells (1 × 10^6^ cells per sample) were washed and equilibrated at 15 °C for 15 min in 500 µl of HBBS (pH = 7.4) containing 1 g/L glucose (HBSS-glucose). An equal volume of NBD-phospholipid in HBSS-glucose was added to the cell suspension (achieving a final concentration of 0.5 µM NBD-PS, 0.2 µM NBD-PC or NBD-PE) following addition of 10 µM glucose conjugates or the respective non-fluorescent phospholipid. After incubating for 15 min at 15 °C, the cell suspensions were placed on ice and the following steps were performed at 4 °C: washed once with ice-cold HBSS, incubation in ice-cold HBSS containing 2% fatty-acid free BSA in order to extract NBD-lipids incorporated into the exoplasmatic leaflet of the plasma membrane, washed once more with ice-cold HBSS and resuspended in HBSS containing 5 mM EDTA. Next, 10.000 cells were immediately analyzed with a FACSCalibur (BD Biosciences) to measure fluorescence of remaining NBD-lipids, reflecting the fraction translocated into the cytoplasmic leaflet of the plasma membrane. The mean of fluorescent intensity per cell was calculated. Propidium iodide–positive cells (i.e., dead cells) were excluded from the analysis.

### Evaluation of particle size by dynamic light scattering

The size of particles was assessed using the dynamic light scattering measurement (Zetasizer, nanoseries Nano-ZS, Malvern, Worcestershire, UK). For the determination, glucose conjugates were diluted with PBS (final concentration of DMSO was 1%) to the concentration as indicated and measurements were immediately performed at 25 °C. Data were analyzed with Zetasizer software V 6.20.

### Transient transfection experiments

HeLa S3 cells were transfected at ~25% confluency. siRNA targeting TMEM30A, ATP8B1 and scrambled were purchased from Dharmacon and 20 µM stocks were prepared in ddH_2_O. Cells were transfected for 48 h with Lipofectamine RNAimax (Invitrogen) using 7.5 nM siRNA. Total RNA was isolated using TRI-Reagent (Zymo Research) followed by chloroform isolation. The reverse transcription was performed using the Verso cDNA Synthesis Kit (Thermo Fisher Scientific) using Anchored Oligo dT primers for cDNA synthesis. Endpoint PCR was performed using Taq DNA Polymerase Master Mix RED (Ampliqon A/S). The PCR was performed as following: initial denaturation at 95 °C for 5 min, 40 cycles for amplification at 95 °C for 30 s, 62 °C for 30 s and 72 °C for 30 s, final elongation at 72 °C for 5 min. Primer sequences were taken as described for ATP8B1^47^, CDC50A and *β*-Actin^48^. For the MGMT assay cells were treated with pre-warmed medium containing 10 µM O^6^BG-Glu for 15 min at 37 °C. Then, the medium was discarded and the cells were washed twice with ice cold PBS. Desorption occurred in ice-cold 1% BSA in PBS for 10 min. The cells were washed in ice cold PBS and then were scrapped off and pelleted for the MGMT assay.

### Statistics

Statistical analysis was performed using GraphPad Prism version 6 (GraphPad Software, La Jolla, CA, USA). A monoexponential function was fitted to the time course of ^3^H-O^6^BTG-Glu uptake by nonlinear regression analysis for graphic presentation. The linear rate of uptake was determined by the slope of accumulation of ^3^H-O^6^BTG-Glu using linear regression analysis. Results are presented as mean +/− standard deviation (SD) or mean +/− standard error (SEM) from at least two independent experiments performed in duplicate. Comparison between samples was performed using the unpaired t test or one-way ANOVA. A p-value of <0.05 was considered as significant.

### Data availability statement

Data published in this paper are available on request.

## Electronic supplementary material

Supplementary Information
