# Supplementary material for: Uptake of glucose-conjugated MGMT inhibitors in cancer cells: role of flippases and type IV P-type ATPases
Source: Sci Rep. 2017 Oct 24;7:13925. doi: 10.1038/s41598-017-14129-x (PMC5655675; doi:10.1038/s41598-017-14129-x)
Supplement: Supplementary file 1 — Supplementary Information [file 41598_2017_14129_MOESM1_ESM.docx]

**Supplementary information:**

**Uptake of glucose-conjugated MGMT inhibitors in cancer cells:**

**role of flippases and type IV P-type ATPases**

Karl-Heinz Tomaszowski^1^, Nadja Hellmann^2^, Viviane Ponath^1^, Hiroyuki Takatsu^3^,Hye-Won Shin^3,4^ and Bernd Kaina^1*^

^1^ Department of Toxicology, University Medical Center, Obere Zahlbacher Strasse 67, D‑55131 Mainz, Germany

^2^ Institute for Molecular Biophysics , Johannes Gutenberg‐University, Jakob Welder Weg 26 D‑55128 Mainz, Germany,

^3^ Graduate School of Pharmaceutical

Sciences, and, Kyoto University, Sakyo-ku, Kyoto 606-8501, Japan

* correspondence: kaina@uni-mainz.de

**Figure S1:** Effect of glucose transporters on O^6^BG-Glu uptake in T98G cells. (A) Time course of the MGMT inhibition of O^6^BG-Glu (25 µM) under different glucose buffer conditions. After indicated time points MGMT activity was determined. (B) Uptake of O^6^BG-Glu (25 µM) was determined in the presence of various glucose transporter inhibitors after 15 min and expressed as percentage of control, defined as O^6^BG-Glu uptake in standard transport buffer without inhibitors. All data represents the mean +/- SD.

**A**

**B**

**Figure S2:** MGMT activity in the presence of MGMT inhibitor O^6^BG-Glucose. Left: HeLa S3 knock-down cells were treated with 10 µM O^6^BG-Glucose for 15 min and MGMT activity was measured afterwards. Right: Knock down efficiency was detected via endpoint PCR for ATP8B1 and TMEM30A (CDC50A). Data are the mean of three independent experiments measured in technical duplicates ± SEM, 1-way ANOVA, Tukey's Multiple Comparison Test, *p < 0.05, **p < 0.01, ***p < 0.001.


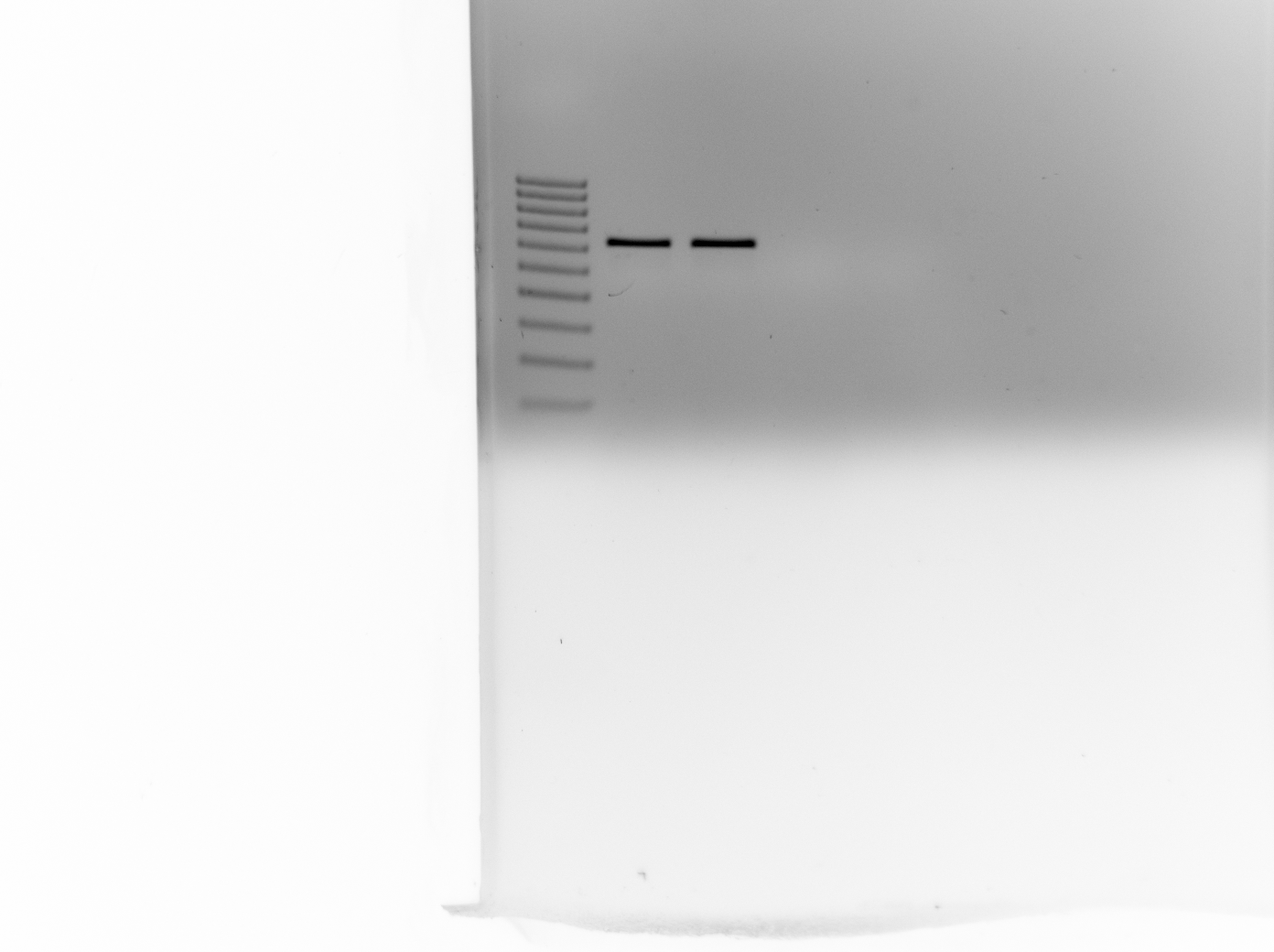


Scr

KD

Con

10 µM

Con

10 µM


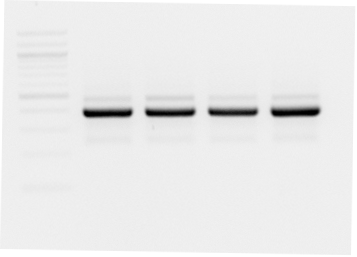


ATP8B1

β-Actin


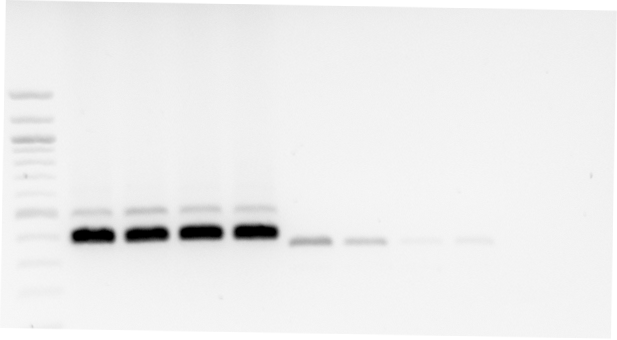

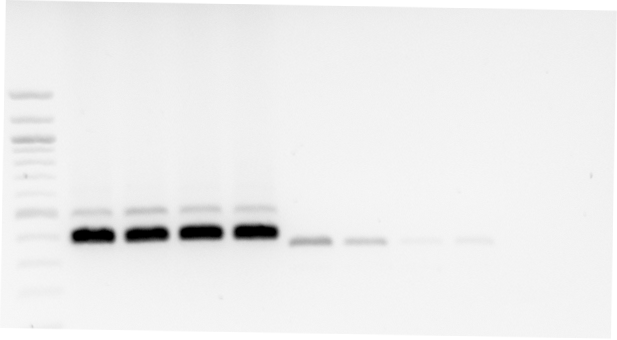


β-Actin

CDC50A
